# Prostate cancer awareness in the city of São Paulo

**DOI:** 10.31744/einstein_journal/2021AO6325

**Published:** 2021-11-19

**Authors:** Feres Camargo Maluf, Felipe Marsiglia Faustino Saporito, Reinolds Amiraldo Corrêa, Pedro Araujo Conesa, Cristiano Linck Pazeto, Leonardo Seligra Lopes, Sidney Glina

**Affiliations:** 1 Centro Universitário FMABC Santo André SP Brazil Centro Universitário FMABC, Santo André, SP, Brazil.

**Keywords:** Knowledge, Prostatic neoplasms, Surveys and questionnaires, Mass screening

## Abstract

**Objective:**

To evaluate awareness of prostate cancer in the population of the city of São Paulo.

**Methods:**

A total of 392 adults were randomly interviewed on public spaces in the city of São Paulo, and answered a questionnaire that addressed demographic questions and specific knowledge about the prostate cancer. A score was used to assess awareness of cancer in general, and of prostate cancer, considering satisfactory knowledge a score of 6 points.

**Results:**

The mean age was 36.9 years (standard deviation of ±12.6) and 58.2% of participants were male. No previous contact with information related to prostate cancer was reported by 45.5% of participants. For these cases, a greater proportion was observed among men aged over 50 years. As to the score, the mean was 3.7 (standard deviation of ±1.3), with a positive correlation among higher scores, higher income and education level. Less than 5% of participants believed they should only search for prostate cancer screening when symptomatic. Finally, among the less frequent responses to risk factors for prostate cancer, is “ethnic origin” (2.8%).

**Conclusion:**

Even though most participants did not have a satisfactory score, the level of awareness demonstrated in this study seems superior to that of other populational series. Hence it suggested the assessed population understood some essential concepts in prostate cancer, such as the importance of screening and the follow-up. The efforts made by the *Sociedade Brasileira de Urologia* on educational campaigns partially explain this. However, working in some concepts, like identifying risk factors for prostate cancer, might optimize screening outcomes.

## INTRODUCTION

Prostate cancer (PCa) is a frequent disease of the middle-aged and older men, being the second most common neoplasm among men and the sixth leading cause of death from malignant neoplasms worldwide.^(1,2)^ In Brazil, PCa has the highest incidence of cancer among men, followed by non-melanoma skin cancer, including in São Paulo (SP), where the estimated incidence is 51.44 per 100 thousand inhabitants.^(3,4)^

An essential strategy in the management of the disease is the early detection, considered as a secondary prevention aiming to detect cancer in earlier stages of development. It includes serum levels of prostate-specific antigen (PSA) and digital rectal exam. However, widespread screening of PCa raised some concerns about “overdiagnosis” and “overtreatment”.^(5)^ Also, no clear benefit in reducing mortality could be established in several studies.^(6,7)^ On the other hand, according to the same studies, with longer follow-up, less patients are required to be treated to prevent death.^(8)^

Thus, controversies in the screening of PCa have generated several recommendations and guidelines worldwide. For instance, the United States Preventive Services Task Force (USPSTF) gave “D” class recommendation on PCa screening, which was more recently changed to class “C” for males between 55 to 69 years old.^(9)^ In contrast, the *Sociedade Brasileira de Urologia* (SBU) recommends a routine screening as from 50 years,^(10)^ following most non-governmental guidelines in Latin America, despite some minor differences.^(5)^

Besides the guidelines, another factor that affects screening is compliance.^(11)^ In Brazil, compliance to the SBU guideline is lower compared to that of the American Urological Association (AUA).^(12)^ The different compliance trends observed in many countries have a multifactorial cause. One of them is patient health literacy, which should be affected by educational campaigns. Few national studies address the population's compliance to the screening guidelines and their level of information.^(13)^

## OBJECTIVE

To evaluate awareness of prostate cancer in the population of the city of São Paulo.

## METHODS

From July 1^st^ to December 7^th^, 2019, randomly selected individuals were submitted to a personal interview in public spaces of the city of São Paulo. More specifically, the public places chosen for the interview were: Paulista Avenue, Subway Station Vila Morumbi, Subway Station Borba Gato, Subway Station Moema, Subway Station Adolfo Pinheiros, and Coach Station Tietê. The target population was adults of both genders (aged >18 years), literate, and currently living in São Paulo. The individuals were approached by a medical student from the *Centro Universitário FMABC*, and invited to answer a questionnaire (Appendix 1). All pieces of information were anonymous to ensure confidentiality, and participants signed an informed consent form. At the end of the interview, the researcher clarified any questions related to PCa raised by the interviewee. The local Research Ethics Committee approved the project (CAAE: 10292419.0.0000.0082).

### Questionnaire description

We developed a three-part self-applicable questionnaire with 26 questions (three open questions and 23 multiple-choice questions). The first part covered demographic and personal characteristics of the participants, while the second addressed specific PCa knowledge. Finally, the last part assessed PCa-related health behavior and screening, only in male participants (Appendix 1).

Based on data from the Brazilian Institute of Geography and Statistics (IBGE - *Instituto Brasileiro de Geografia e Estatística*), an economic stratification was based on minimum wage (R$ 937,00 or US$ 245.51).^(14)^ The dollar exchange rate used was from the beginning of the interview, *i.e*., 1.00 USD was equal to 3.8165 Brazilian *Reals*.^(15)^ The order of the questions was determined to avoid bias in answers, and the order of the multiple-choice options, which were alphabetical, except the options “I do not know the answer” and “Others,” both at the end of the alternatives.

### Score

We created an accuracy index considering only the eight questions on knowledge about PCa. Each correct answer generated a point (total of eight points). A score greater than or equal to six points was considered appropriate awareness. The score was designed to prevent participants from answering correctly by chance.

### Data analysis

Considering a population of approximately ten million inhabitants (IBGE census),^(16)^ an alpha value of 5% and a 95% confidence interval, a sample size of 384 was estimated. In the descriptive analysis, continuous variables were presented as means and standard deviation (SD), and categorical variables as frequency and percentages (%). Each question was individually analyzed to determine the missing data (empty cells or uninterpretable answers). For questions that precisely assess knowledge of PCa (including those of personal and family history), all empty cells and uninterpretable answers were grouped with the alternative “I do not know/I do not know the answer” (*e.g*., more than one answer in a multiple-choice question). For each question analysis, a critical value of 10% was established for missing data.

We also conducted an exploratory analysis, comparing proportions of answers and means of scores, according to demographic variables. Moreover, scores were correlated with the presence of a family history of cancer, previous contact with PCa information, means of reaching PCa information, and participant´s occupation (healthcare worker or not). Only the most frequent answer(s) and accurate answer(s) were chosen to be analyzed, while the others were gathered and defined as “Others” in this secondary analysis. Some demographic characteristics were categorized to summarize the results. For proportions, the comparisons were made through chi-square test χ^2^, whereas for scores, an unpaired *t*-test was applied. Values of p<0.05 were considered statistically significant. The data analysis was performed in (SPSS), version 21 (IBM Software^®^).

## RESULTS

### Demographics

A total of 392 participants were included. Overall missing data was 1.4% – none presenting more than the critical value of 10%. We summarized patients’ demographic characteristics in table 1. The mean age and SD were 36.9±12.6 years, and 58.2% of participants were male. The majority of participants (86.9%) had complete high school or further graduation, and had an income ranging from 0 to R$ 9.370,00/US$ 2,455.10 (87.1%). In addition, 98.2% of interviewees had no past history of previous cancer, while 50% had a positive family history. Breast, prostate, and skin cancer (10.9%, 6.89%, and 6.37%, respectively) were the most prevalent types (personal or family history).

**Table 1:** Summary of the sample's demographic characteristics

Variable		
Age, years		36.9±12.6
Sex		
	Male	228/392 (58.2)
	Female	164/392 (41.8)
Income, US$		
	0-491.02	104/389 (26.7)
	491.03-982.04	125/389 (32.1)
	982.05-2,455.10	110/389 (28.3)
	2,455.11-4,910.20	31/389 (8.0)
	≥4,910.21	19/389 (4.9)
Educational level		
	Graduate	79/390 (20.3)
	Further education	139/390 (35.6)
	Complete high school	121/390 (31.0)
	Incomplete high school	29/390 (7.4)
	Complete elementary school	9/390 (2.3)
	Incomplete elementary school	13/390 (3.3)
Marital status		
	Single	215/391 (54.8)
	Married	134/391 (34.2)
	Divorced	22/391 (5.6)
	Consensual marriage	14/391 (3.6)
	Widow/er	6/391 (1.5)
Race		
	White	238/391 (60.9)
	Brown (*Pardo*)	96/391 (24.6)
	*Mulatto*	6/391 (1.5)
	Asian	14/391 (3.6)
	Black	37/391 (9.5)
Healthcare professional		56/392 (14.3)
Personal diagnosis of cancer		7/391 (1.8)
Diagnosis of cancer in the family		204/390 (52.3)

Results expressed as mean±standard deviation or n/total n (%).

### Information on prostate cancer

Approximately 45% of participants reported never having had contact with PCa information. For these cases, a higher proportion of older males (age >50 years) was noted (Table 2). As to information sources, the most reported were healthcare professionals, internet/social media, and university/school. Individuals older than 50 years reported more frequently healthcare professional as the information source (75.8% and 38.1%, for >50 years and ≤50 years, respectively; p<0.001). The primary sources of information for men were healthcare professionals and internet/social media. Higher educational levels were associated with the use of internet/social media (p=0.002).

**Table 2:** Answer analysis regarding the previous contact with prostate cancer accordingly to demographic characteristics

Variable		No (178/391)	Yes (213/391)	p value
Age, years				0.010
	≤50	26.7	73.3	
	>50	48.3	51.7	
Sex				0.001
	Male	38.3	61.7	
	Female	55.5	44.5	
Income, US$				0.127
	0-491.02	51.9	48.1	
	491.03-982.04	40.0	60.0	
	982.05-2,455.10	50.5	49.5	
	2,455.11-4,910.20	32.3	67.7	
	≥4,910.21	36.8	63.2	
Educational level				0.283
	Graduate	35.4	64.6	
	Further education	46.4	53.6	
	Complete high school	52.9	47.1	
	Incomplete high school	44.8	55.2	
	Complete elementary school	44.4	55.6	
	Incomplete elementary school	38.5	61.5	

Results expressed as %.

### Score

Scores obtained ranged from zero to eight, with a mean of 3.7 (±1.3). Participants with an income between zero to R$ 1.874,00/US$ 491.02 had lower scores (3.3±1.4) compared to those with higher income (4.0±0.9). Moreover, the higher the educational level, the higher the scores (Table 3). The mean score value of healthcare professionals was similar to that of other interviewees (3.9±1.0 *versus* 3,6±1,4; p=0.182). The previous contact with PCa information and source of information was not correlated with the score (p=0.651) (Table 3).

**Table 3:** Comparison of scores accordingly to demographic characteristics

Variable		n	Score accuracy (mean±SD)	p value
Age, years				0.797
	≤50	345	3.7±1.3	
	>50	45	3.6±1.4	
Sex				0.257
	Male	228	3.6±1.4	
	Female	164	3.7±1.3	
Income, US$				
	0-491.02	104	3.3±1.4*†	0,020
	491.03-982.04	125	3.7±1.3	0,023*
	982.05-2,455.10	110	3.9±1.3†	0,088†
	2,455.11-4,910.20	31	4.0±0.9*	
	≥4,910.21	19	3.6±1.5	
Educational level				<0,001
	Graduate	79	3.8±1.1‡§	0,019‡
	Further education	139	3.9±1.3¶&	0,012§
	Complete/Incomplete high School	150	3.4±1.3‡¶#	0,001¶
	Complete/Incomplete elementary school	22	2.8±1.7§&#	<0,001&
Searched/received information related to PCa				0,041#
	No	178	3.6±1,4	0.651
	Yes	213	3.7±1,3	
Healthcare professional				
	No	336	3.6±1.4	0.182
	Yes	56	3.9±1.0	
Family history of cancer				
	No	186	3.6±1.3	0.184
	Yes	204	3.7±1.4	

* comparison between income ranges US$ 0 to US$ 491.02 and US$ 2,455.11 to US$ 4,910.20

† comparison between income ranges US$ 0 to US$ 491.02 and US$ 982.05 to US$ 2,455.10

‡ comparison between educational levels graduate and complete/incomplete high school

§ comparison between educational levels graduate and complete/incomplete elementary school

¶ comparison between educational levels further education and complete/incomplete high school

& comparison between educational levels further education and complete/incomplete elementary school

# comparison between educational levels complete/incomplete high school and complete/incomplete elementary school.

SD: standard deviation; PCa: prostate cancer.

### Specific knowledge

More than 50% answered that the most common types of cancer are prostate in men, and breast in women. Furthermore, the most frequently reported risk factors for PCa were positive family history (28.7%), age (16.9%), smoking (13.3%), I do not know (9.9%), and alcohol consumption (8.1%). Less frequent answers included obesity (4.3%) and ethnic origin (2.8%).

The most reported factors related to better PCa outcomes were undergo routine blood exams (29.8%), physical exercise (24.5%), eat fruits and vegetables (17.4%), I do not know (12.1%), and body weight control (9.5%).

Approximately 82.9% answered they should be submitted to PCa screening, even when asymptomatic. Some differences were noted among age groups. More specifically, assuming an asymptomatic scenario, 70% of subjects aged ≤50 years believed that they should seek medical assistance before the age of 50, while 7.2% of the same age group believed that they should seek medical assistance between 30 and 50 years of age. In contrast, the proportion of answers for the same questions was 57.8% and 24.4% among those aged >50 years (p=0.008).

Most individuals (66.6%) answered they should perform the screening annually, while 2% believed they did not require regular follow-up, since the initial assessment was enough. Regarding the possible diagnostic tools for PCa, the most frequent answers were digital rectal exam (44.5%), blood test/PSA levels (29.7%), and prostate ultrasonography (16.4%).

### Male health behavior toward screening exams for prostate cancer

Most participants reported never having sought PCa screening (68.3%), followed by 16.3% who reported having recently visited a physician for prostate-related exams. Most men reported never having had PSA (72%) or digital rectal exam (85%). In a sub-analysis, participants over 50 years of age reported more frequently, never seeking screening (78.2% *versus* 3.4%, for >50 years and ≤50 years; p<0.001) (Table 4).

**Table 4:** Answers analysis, according to demographic characteristics, regarding the last time male participants sought prostate cancer screening (question 19 of appendix 1)

Variable		Never (155/227)	>5 years (5/227)	3-5 years (6/227)	1-2 years (24/227)	This year (37/227)	p value
Age, years							<0.001
	≤50	3.4	3.4	6.9	44.8	41.4	
	>50	78.2	2.0	2.0	5.6	12.2	
Income, US$							0.145
	0-491.02	76.9	1.5	3.1	4.6	13.8	
	491.03-982.04	75.4	1.4	2.9	13.0	7.2	
	982.05-2,455.10	61.7	3.3	3.3	10.0	21.7	
	2,455.11-4,910.20	40.0	5.0	0.0	25.0	30.0	
	≥4,910.21	63.6	0.0	0.0	9.1	27.3	
Educational level							0.077
	Graduate	65.8	2.6	0.0	7.9	23.7	
	Further education	63.0	2.5	2.5	8.6	23.5	
	Complete high school	78.9	1.4	4.2	9.9	5.6	
	Incomplete high school	60.0	0.0	5.0	15.0	20.0	
	Complete elementary school	100.0	0.0	0.0	0.0	0.0	
	Incomplete elementary school	58.3	8.3	0.0	33.3	0.0	

Results expressed as %.

Among the reasons for never having done the digital rectal exam, the most frequent ones were I am not old enough (63.5%), I would do it without problems (38.6%) and it does not have to be done (20.1%). Among older men, 75% responded the physician decided not to do so.

## DISCUSSION

The study's mean score value was 3.4, probably reflecting an insufficient knowledge in the considered population. Further, it might suggest an issue in educational campaign effectiveness, especially among those with the proper age for PCa screening.

Almost half the participants reported they had never had contact with PCa information, especially the older individuals. The fact that more younger participants use the internet and social media as an informative tool, whereas older participants rely more on a healthcare professional approach (less present in the daily routine), could partially explain this. Increasing the contact between the healthcare professional and older patients via different platforms, such as applications or telemedicine, could be an option.

A different finding of the study, however, is that among 178 participants who responded that they never had had contact with PCa screening information, 162 answered digital rectal exam or PSA, as the primary diagnostic tools for PCa. Also, the mean score between participants with or without previous contact with PCa information was similar. Therefore, these answers challenged our previous suspicions, elaborated in the beginning of the discussion, of ineffectiveness in disclosing information to the Brazilian public. Some participants may not have enough information to feel comfortable about knowledge of the disease, which is essential in a shared decision considering the benefits and risks of PCa screening. A population with more consistent awareness would be more compliant with the recommendations.

In the Brazilian scenario, few similar studies have assessed this topic. In a previous study by Ribeiro et al., including 30 males, the proportion of respondents performing PSA was higher than ours.^(17)^ However, 80% of Ribeiro et al., patients had a personal history of cancer, imposing a consistent difference in sample characteristics. Controversially, 40% of sample was unaware of PCa screening, while only 17% were in our sample.

In another study involving 160 individuals,^(18)^ 63.8% reported PSA and digital rectal exam as a diagnosis tool for PCa (similarly to 74.2% of present study); 40.6% considered annual screening necessary for PCa (less than 66.6% of present study).

Previous studies enrolled only male participants and had different questionnaires compositions, making comparisons with the present study difficult. The population of the city of São Paulo has the highest educational and income levels among Brazilian states, which is correlated with higher PCa awareness.^(19)^ Even though our studied population presented some gaps of knowledge, such as few participants identified ethnic origin as a risk factor for PCa. These identified gaps altogether could be addressed in future education campaigns.^(2,20)^

According to Allen et al.,^(21)^ in a stable relationship, women have an essential role in seeking and disseminating information related to PCa to their male partners. Therefore, it is crucial to assess the women's awareness of PCa to understand adherence to screening recommendations better. Among our study responders, a higher proportion of females did not seek information on PCa, which is a potential issue to improve SBU campaign results.^(13)^

In the international scenario, there are several studies on the same topic. A study from Burkina Faso demonstrated insufficient knowledge on PCa, since 62% of participants had never seen the terms “prostate” or “ prostate cancer”.^(2)^ Those findings were similar in other studies from Nigeria.^(22,23)^

A similarly designed international survey, involving European countries and the United States, demonstrated higher knowledge levels compared to studies from African countries. Especially in the United States, 97% of participants were aware of the PCa.^(24)^ However, 50% of participants were unaware of the diagnostic tools for PCa, compared to 2.4% of our studied sample. In addition, 1% of participants in the international survey was not aware the disease could be asymptomatic, whereas 82.9% of our participants knew they should seek PCa diagnosis regardless of symptoms. Likewise, our study and the international research reported greater awareness of breast cancer than PCa, suggesting the influence of educational campaigns on public awareness, as educational programs on breast cancer are more established. Also, both studies demonstrated similar percentages in risk factors correlated with PCa – age and positive family history were mentioned in 73% and 44% of the answers, compared to 28.7% and 16.9% of our answers, respectively. Ethnic origin was not frequently answered in both studies.

The actual study indirectly assessed PCa awareness by asking the most incident type of cancer among males and females; the participants more frequently answered breast cancer as the most incident when compared to PCa.

The different knowledge levels between the present study and the international survey raise questions regarding screening recommendations and screening adherence. The population of the city of São Paulo knows enough to understand the risk and benefits of PCa screening for an individual decision making, as recommended by the USPSTF? In the United States, findings demonstrate a lower search for PSA, following the national recommendations, which could be beneficial in reducing unnecessary biopsies and invasive treatments.^(25)^ However, evidence from the actual study suggests that some male individuals at a higher risk of developing PCa are already receiving less screening.^(26,27)^ A possibility is that this population is unaware of some critical components of the disease to decide if they would have more benefits or not, undergoing a screening program.

Pazeto et al., demonstrated that amongst the participants aged under 40 years submitted to PSA testing, most of them only did it as a health check-up since they had no clear indication of it.^(28)^ Therefore, many participants do not specifically seek PCa screening, but they probably do it as a recommendation of their healthcare provider or as a general routine health check-up.^(28)^ Thus, it might be challenging to establish an individual decision as a recommendation for PCa screening, if the individuals do not even know what they are submitted to.

One of the limitations of the study is the population enrolled may be not fully representative of the target population. An example is that only 3.3% of population studied did not complete elementary school, compared to 35.03% of the population within this educational level of the city of São Paulo, in the 2010 census.^(29)^ Despite that, the population ethnicities were similar in our study to the real percentage of the city's population (for instance, white group population of study 60.9% *versus* 60.65% white group population of São Paulo, SP, Brazil).^(30)^ Moreover, the study addressed a younger population compared to the normally affected by PCa; however, it is important to understand the knowledge of this specific group to prepare strategies and increase their awareness in the future.

Another limitation was the use of a non-validated questionnaire, hindering comparison with similar studies. It does not directly approach awareness of the population related to the SBU screening guidelines, although being able to indirectly demonstrate it, by asking when they should visit the doctor for PCa screening. Although there is not a proved protective factor, as it is written in the questionnaire (Appendix 1), the question assessed the health behavior towards PCa, by asking, as an example, if they visited the doctor routinely for PCa screening exams, as recommended by the SBU. The design of the score could also cause some imprecisions when interpreting the results, since it tries to assess knowledge of the participants in an arbitrary way.

## CONCLUSION

Even though most participants did not achieve a satisfactory score, the awareness level shown in this study seems superior to other population series. Thus, it suggests the assessed population understood some essential concepts in prostate cancer, such as the importance of screening and the follow-up. The efforts of the *Sociedade Brasileira de Urologia* towards educational campaigns partially explain this. Nonetheless, working in some concepts, like identifying risk factors for prostate cancer, might optimize screening outcomes.

## Appendix 1 Questionnaire knowledge evaluation

**Table:**

1. What is your sex?	
	Female
	Male
2. What is your age? ____________________	
3. What is your monthly income?	
	a. ≥$ 4,910.21
	b. $ 2,455.11 to $ 4,910.20
	c. $ 982.05 to $ 2,455.10
	d. $ 491.03 to $ 982.04
	e. $ 0 to $ 491.02
4. What is your educational level?	
	a. Incomplete elementary school
	b. Complete elementary school
	c. Incomplete high school
	d. Complete high school
	e. Further education
	f. Graduate
5. What is your marital status?	
	a. Single
	b. Married
	c. Divorced
	d. Consensual marriage
	e. Widow/er
6. What is your race?	
	a. White
	b. Brown (*Pardo*)
	c. Mulatto
	d. Asian
	e. Black
	f. Other: __________________
7. Are you a healthcare professional?	
	a. Yes
	b. No
7A. If yes, in which healthcare field?	
	a. Medicine
	b. Physiotherapy
	c. Occupational Therapy
	d. Physical Education
	e. Pharmacy
	f. Audiology and Speech therapy
	g. Nutrition
	h. Dentistry
	i. Psychology
	j. Chiropractic care
	k. Other: __________________
8. Have you ever been diagnosed with any type of cancer?	
	a. Yes
	b. No
	8A. If yes, which type? ____________________
9. Any close relatives (consider only grandfathers/grandmothers, father/mother, sons/daughters, and brothers/sisters) have ever been diagnosed with any type of cancer?	
	a.Yes
	b. No
9A. If yes, which type of cancer? ____________________	
10. What is the most common type of cancer among men?	
	a. Mouth cancer
	b. Esophageal cancer
	c. Gastric cancer
	d. Liver cancer
	e. Intestinal cancer
	f. Breast cancer
	g. Pancreas cancer
	h. Skin cancer*
	i. Penile cancer
	j. Prostate cancer
	k. Lung cancer
	l. Kidney cancer
	m. I do not know the answer
	n. Other: __________________
	*Correct answer: skin cancer (h)
11. What is the most common type of cancer among women?	
	a. Mouth cancer
	b. Esophageal cancer
	c. Gastric cancer
	d. Liver cancer
	e. Intestinal cancer
	f. Breast cancer
	g. Pancreas cancer
	h. Skin cancer*
	i. Penile cancer
	j. Prostate cancer
	k. Lung cancer
	l. Kidney cancer
	m. I do not know the answer
	n. Other: __________________
	*Correct answer: skin cancer (h)
12. Have you ever searched or received prostate cancer information?	
	a. Yes
	b. No
12A. If yes, where did you search or receive prostate cancer information? (you can choose more than one option)	
	a. School/university
	b. Workplace
	c. Church/religious cult
	d. Newspaper/magazine
	e. Internet/social media
	f. Physician/other healthcare professional
	g. Friends
	h. Family
	i. Other: __________________
13. Prostate cancer affects which genders?	
	a. Both genders
	b. Male*
	c. Female
	d. I do not know the answer
	*Correct answer: male (b)
14. Which of the factors below are prostate cancer risk factors? (you can choose more than one option)	
	a. Family history of cancer*
	b. Stress
	c. Alcohol use
	d. Lack of hygiene
	e. Age*
	f. Masturbation
	g. Socioeconomical range
	h. Obesity*
	i. Pollution
	j. Race/ethnicity*
	k. Frequent sexual relation
	l. Smoking
	m. I do not know the answer
	n. Other: __________________
	*Correct aswers: family history of cancer (a); age (e); obesity (h); race/ethnicity (j)
15. Which of the factors below can prevent or decrease prostate cancer risk? (you can choose more than one option)	
	a. Eat red meat every week
	b. Eat fruits and vegetables*
	c. Physical exercise*
	d. Routine blood tests*
	e. Body weight control*
	f. Do not keep the mobile phone in your pocket
	g. Avoid many sexual relations
	h. Do not masturbate
	i. Use sun protector
	j. I do not know the answer
	k. Other: __________________
	*Correct answers: eat fruits and vegetables (b); physical exercise (c); undergo routine blood tests (d); body weight control (e)
16. When should you seek medical care related to prostate cancer?	
	a. Only when presenting correlated symptoms (example: blood in urine)
	b. Asymptomatic, between 30 to 40 years
	c. Asymptomatic, between 41 to 50 years*
	d. Asymptomatic, between 51 to 60 years
	e. Asymptomatic, between 61 to 70 years
	f. I do not know the answer
	g. Other: __________________
	*Correct answer: asymptomatic, between 41 to 50 years (c)
17. How often should you return to the physician for prostate cancer monitoring?	
	a. Every 2 years
	b. Every 3 years
	c. Every 4 years
	d. Every 5 years
	e. Annually (every year)*
	f. No need to return if the exams are normal
	g. I do not know the answer
	h. Other: __________________
	*Correct answer: annually (e)
18. What exams are needed in the initial prostate cancer investigation? (you can choose more than one option)	
	a. Digital rectal exam*
	b. Nuclear magnetic resonance
	c. Blood test/PSA*
	d. Urine test
	e. Bone test
	f. Prostate ultrasonography
	g. I do not know the answer
	*Correct answers: digital rectal exam (a); blood test/PSA (c)
19. QUESTION ONLY FOR MEN – When was the last time you saw a physician for prostate-related exams?	
	a. This year
	b. 1 year ago
	c. 2 years ago
	d. 3 years ago
	e. 4 years ago
	f. 5 years ago
	g. More than 5 years ago
	h. Never
20. QUESTION ONLY FOR MEN – Have you ever done blood exam for prostate cancer? (PSA dosage exam – prostatic specific antigen)?	
	a. Yes
	b. No
21. QUESTION ONLY FOR MEN – Have you ever done the digital rectal exam?	
	a. Yes
	b. No
21A. QUESTION ONLY FOR MEN – If you did not do the digital rectal exam, why you did not do it? (you can choose more than one option)	
	a. It interferes in anal or rectal anatomy/physiology
	b. It interferes sexual potency
	c. It interferes sexuality (sexual interest)
	d. It is painful
	e. Physician did not request it
	f. Fear
	g. I am not old enough
	h. It does not need to be done
	i. I have no worries. I would do it, it is no problem
