# Supplementary material for: Prostate cancer awareness in the city of São Paulo
Source: Einstein (Sao Paulo). 2021 Nov 19;19:eAO6325. doi: 10.31744/einstein_journal/2021AO6325 (PMC8664284; doi:10.31744/einstein_journal/2021AO6325)
Supplement: Translation

# Translation: Conhecimento da população da cidade de São Paulo a respeito do câncer de próstata

**Authors:** Feres Camargo Maluf, Felipe Marsiglia Faustino Saporito, Reinolds Amiraldo Corrêa, Pedro Araujo Conesa, Cristiano Linck Pazeto, Leonardo Seligra Lopes, Sidney Glina

**Affiliations:** 1 Santo André SP Brasil Centro Universitário FMABC, Santo André, SP, Brasil.

**Keywords:** Conhecimento, Neoplasias da próstata, Inquéritos e questionários, Programas de rastreamento

## Abstract

**Objetivo:**

Avaliar o conhecimento da população da cidade de São Paulo em relação ao câncer de próstata.

**Métodos:**

Foram entrevistados randomicamente 392 adultos em espaços públicos da cidade de São Paulo, os quais responderam a um questionário que abordava questões demográficas e de conhecimentos específicos sobre o câncer de próstata. Um escore foi utilizado para avaliar o conhecimento de câncer em geral e do câncer de próstata, considerando um conhecimento satisfatório com escore de 6 pontos.

**Resultados:**

A média de idade foi de 36,9 anos (desvio-padrão de ±12,6), e 58,2% dos participantes eram do sexo masculino. Ausência de contato anterior com informações relacionadas ao câncer de próstata foi relatada por 45,5% dos participantes. Nesses casos, maior proporção foi observada entre os homens com mais de 50 anos. Quanto ao escore, a média foi 3,7 (desvio-padrão de ±1,3), com correlação positiva entre maiores escores e maiores renda e escolaridade. Menos de 5% dos participantes acreditavam que só deveriam procurar o rastreamento do câncer de próstata quando sintomáticos. Por fim, entre as respostas menos frequentes aos fatores de risco para câncer de próstata, encontrou-se “etnia” (2,8%).

**Conclusão:**

Embora a maioria dos participantes não tenha apresentado escore satisfatório, o nível de conhecimento revelado neste estudo parece superior ao de outros estudos populacionais. Assim, sugere-se que a população avaliada tenha compreendido alguns conceitos essenciais do câncer de próstata, como a importância do rastreamento e do acompanhamento. Os esforços da Sociedade Brasileira de Urologia nas campanhas educacionais explicam parcialmente isso. No entanto, trabalhar em alguns conceitos, como identificar fatores de risco para câncer de próstata, pode otimizar os resultados do rastreamento.

## INTRODUÇÃO

O câncer de próstata (CaP) é uma doença frequente em homens de meia-idade e mais velhos, sendo a segunda neoplasia mais comum entre os homens e a sexta causa de morte por neoplasias malignas em todo o mundo.^(1,2)^ No Brasil, o CaP tem a incidência mais alta de câncer entre os homens, seguido pelo câncer de pele não melanoma, inclusive em São Paulo (SP), onde a incidência estimada é de 51,44 por 100 mil habitantes.^(3,4)^

Uma estratégia essencial no manejo da doença é a detecção precoce, considerada uma prevenção secundária que visa detectar o câncer em estágios mais iniciais de desenvolvimento. A detecção precoce inclui dosagem sérica de antígeno específico da próstata (PSA) e toque retal. No entanto, o rastreamento generalizado de CaP levantou algumas preocupações sobre “excesso de diagnósticos” e “tratamento excessivo”.^(5)^ Além disso, em diversos estudos nenhum benefício evidente foi determinante na redução da mortalidade.^(6,7)^ Por outro lado, de acordo com os mesmos estudos, com o aumento do seguimento, é necessário um menor número de pacientes para prevenir óbito.^(8)^

Dessa forma, as controvérsias no rastreamento de CaP geraram várias recomendações e diretrizes em todo o mundo. Por exemplo, a *U.S. Preventive Services Task Force* (USPSTF) deu recomendação classe “D” para o rastreamento de CaP, que foi mais recentemente alterada para classe “C” para homens entre 55 e 69 anos de idade.^(9)^ Por outro lado, a Sociedade Brasileira de Urologia (SBU) recomenda rastreamento de rotina a partir dos 50 anos,^(10)^ seguindo a maioria das diretrizes não governamentais da América Latina, apesar de algumas pequenas diferenças.^(5)^

Além das diretrizes, outro fator que afeta o rastreamento é a adesão.^(11)^ No Brasil, a adesão à diretriz da SBU é menor em relação à da *American Urological Association* (AUA).^(12)^ A causa das diferentes tendências de adesão observadas em muitos países é multifatorial. Um dos fatores é o letramento em saúde, que deve ser afetado por campanhas educativas. Poucos estudos nacionais abordam a adesão da população às diretrizes de rastreamento e o grau de informação.^(13)^

## OBJETIVO

Avaliar o conhecimento da população da cidade de São Paulo em relação ao câncer de próstata.

## MÉTODOS

De 1º de julho a 7 de dezembro de 2019, foram realizadas entrevistas com pessoas escolhidas aleatoriamente em espaços públicos da cidade de São Paulo. Os locais públicos designados para entrevistas foram mais especificamente os seguintes: Avenida Paulista, Estação do Metrô Vila Morumbi, Estação do Metrô Borba Gato, Estação do Metrô Moema, Estação do Metrô Adolfo Pinheiros e Terminal Rodoviário Tietê. A população-alvo eram adultos de ambos os sexos (idade >18 anos), alfabetizados e atualmente residindo em São Paulo. Os indivíduos foram abordados por um estudante de medicina do Centro Universitário FMABC e convidados a responder um questionário (Apêndice 1). Todas as informações eram anônimas para garantir a confidencialidade, e os participantes assinaram um Termo de Consentimento Livre e Esclarecido. Ao final da entrevista, o pesquisador esclareceu quaisquer questões relacionadas ao CaP trazidas pelo entrevistado. O Comitê de Ética em Pesquisa local aprovou o projeto (CAAE: 10292419.0.0000.0082).

### Descrição do questionário

Foi desenvolvido um questionário autoaplicável em três partes, com 26 perguntas (três perguntas abertas e 23 de múltipla escolha). A primeira parte abordou características demográficas e pessoais dos participantes e a segunda, conhecimentos específicos sobre CaP. A última parte, apenas para participantes homens, avaliava o comportamento de saúde relacionado a CaP e rastreamento (Apêndice 1).

Com base em dados do Instituto Brasileiro de Geografia e Estatística (IBGE), a estratificação econômica foi baseada no salário mínimo (R$ 937,00 ou US$ 245.51).^(14)^ A cotação do dólar usada foi a do período do início da entrevista, ou seja, US$ 1.00 equivalia a R$ 3.8165.^(15)^ A ordem das perguntas foi determinada para evitar viés nas respostas. O mesmo foi feito para as opções de múltipla escolha, estando em ordem alfabética, com exceção das opções “Eu não sei a resposta” e “Outras”, ambas no final das alternativas.

### Escore

Foi criado um índice de precisão considerando apenas as oito perguntas relacionadas ao conhecimento sobre CaP. Cada resposta correta gerava um ponto (total de oito pontos). Um escore maior ou igual a seis pontos foi considerado como conscientização adequada. O escore foi projetado para evitar que os participantes respondessem corretamente por acaso.

### Análise dos dados

Considerando uma população de aproximadamente 10 milhões de habitantes segundo censo do IBGE,^(16)^ valor alfa de 5% e intervalo de confiança de 95%, foi estimado tamanho amostral de 384 indivíduos. Na análise descritiva, as variáveis contínuas foram apresentadas como médias e desvio-padrão (DP) e as variáveis categóricas, como frequência e percentagem (%). Cada pergunta foi analisada individualmente para determinar os dados perdidos (células vazias ou respostas não interpretáveis). Para perguntas que avaliavam precisamente o conhecimento sobre CaP (incluindo as sobre história pessoal e familiar), todas as células vazias e respostas não interpretáveis foram agrupadas na alternativa “Eu não sei/não sei a resposta” (por exemplo: mais de uma resposta em uma pergunta de múltipla escolha). Para cada análise de pergunta, foi estabelecido valor crítico de 10% para dados perdidos.

Também foi conduzida uma análise exploratória, comparando proporções de respostas e médias de escores, segundo variáveis demográficas. Os escores foram ainda correlacionados com presença de história familiar de câncer, contato prévio com informações sobre CaP, formas de acessar informações sobre CaP e ocupação do participante (profissional de saúde ou não). Apenas as respostas mais frequentes e precisas foram escolhidas para análise, enquanto as demais foram coletadas e definidas como “Outras” nessa análise secundária. Algumas características demográficas foram categorizadas para resumir os resultados. Para as proporções, as comparações foram feitas por meio do teste de χ2, enquanto, para escores, foi aplicado teste *t* não pareado. Os valores de p<0,05 foram considerados estatisticamente significativos. A análise dos dados foi realizada no programa (SPSS), versão 21 (IBM Software^®^).

## RESULTADOS

### Resultados demográficos

Foram incluídos 392 participantes. O total de dados perdidos foi de 1,4% – nenhum apresentando mais do que o valor crítico de 10%. Resumimos as características demográficas dos pacientes na tabela 1. A média de idade e o DP foram 36,9±12,6 anos, e 58,2% dos participantes eram homens. A maioria dos participantes (86,9%) possuía ensino médio completo ou ensino superior, e a renda variava de zero a R$ 9.370,00/ US$ 2,455.10 (87,1%). Além disso, 98,2% dos entrevistados não tinham história pregressa de câncer, enquanto 50% tinham história familiar positiva. Câncer de mama, próstata e pele (10,9%; 6,89% e 6,37%, respectivamente) foram os tipos mais prevalentes, segundo história pessoal ou familiar.

**Tabela 1:** Resumo das características demográficas da amostra

Variável		
Idade, anos		36,9±12,6
Sexo		
	Masculino	228/392 (58,2)
	Feminino	164/392 (41,8)
Renda, US$		
	0-491.02	104/389 (26,7)
	491.03-982.04	125/389 (32,1)
	982.05-2,455.10	110/389 (28,3)
	2,455.11-4,910.20	31/389 (8,0)
	≥4,910.21	19/389 (4,9)
Escolaridade		
	Pós-graduação	79/390 (20,3)
	Graduação	139/390 (35,6)
	Ensino médio completo	121/390 (31,0)
	Ensino médio incompleto	29/390 (7,4)
	Fundamental completo	9/390 (2,3)
	Fundamental incompleto	13/390 (3,3)
Estado civil		
	Solteiro/a	215/391 (54,8)
	Casado/a	134/391 (34,2)
	Divorciado/a	22/391 (5,6)
	União estável	14/391 (3,6)
	Viúvo/a	6/391 (1,5)
Raça		
	Branca	238/391 (60,9)
	Parda	96/391 (24,6)
	Mulata	6/391 (1,5)
	Asiática	14/391 (3,6)
	Negra	37/391 (9,5)
Profissional da saúde		56/392 (14,3)
Diagnóstico pessoal de câncer		7/391 (1,8)
Diagnóstico familiar de câncer		204/390 (52,3)

Resultados expressos como média±desvio-padrão ou n/n total (%).

### Informação sobre câncer de próstata

Cerca de 45% dos participantes relataram nunca ter tido contato com informações sobre CaP. Para esses casos, observou-se proporção maior de homens mais velhos (idade >50 anos) (Tabela 2). Quanto às fontes de informação, as mais relatadas foram profissionais da saúde, internet/mídia social e universidade/escola. Indivíduos acima de 50 anos relataram com mais frequência como fonte de informação, profissionais da saúde (75,8% e 38,1%, para >50 anos e ≤50 anos, respectivamente; p<0,001). As principais fontes de informação entre os homens foram profissionais da saúde e internet/mídia social. Maior escolaridade estava associada ao uso de internet/mídia social (p=0,002).

**Tabela 2:** Análise das respostas quanto a contato prévio com câncer de próstata conforme as características demográficas

Variável		Não (178/391)	Sim (213/391)	Valor de p
Idade, anos				0,010
	≤50	26,7	73,3	
	>50	48,3	51,7	
Sexo				0,001
	Masculino	38,3	61,7	
	Feminino	55,5	44,5	
Renda, US$				0,127
	0-491.02	51,9	48,1	
	491.03-982.04	40,0	60,0	
	982.05-2,455.10	50,5	49,5	
	2,455.11-4,910.20	32,3	67,7	
	≥4,910.21	36,8	63,2	
Escolaridade				0,283
	Pós-graduação	35,4	64,6	
	Graduação	46,4	53,6	
	Ensino médio completo	52,9	47,1	
	Ensino médio incompleto	44,8	55,2	
	Fundamental completo	44,4	55,6	
	Fundamental incompleto	38,5	61,5	

Resultados expressos como %.

### Escore

Os escores obtidos variaram de zero a oito, com média de 3,7 (±1,3). Os participantes com renda entre zero e R$ 1.874,00/US$ 491.02 tiveram escores mais baixos (3,3±1,4) comparados àqueles com maior renda (4,0±0,9). Além disso, quanto maior a escolaridade, maiores os escores (Tabela 3). O escore médio para os profissionais da saúde foi semelhante ao de outros entrevistados (3,9±1,0 *versus* 3,6±1,4; p=0,182). O contato prévio com informações sobre CaP e a fonte de informação não apresentaram correlação com o escore (p=0,651) (Tabela 3).

**Tabela 3:** Comparação dos escores de acordo com as características demográficas

Variável		n	Precisão do escore (média±DP)	Valor de p
Idade, anos				0,797
	≤50	345	3,7±1,3	
	>50	45	3,6±1,4	
Sexo				0,257
	Masculino	228	3,6±1,4	
	Feminino	164	3,7±1,3	
Renda, US$				
	0-491.02	104	3,3±1,4*†	0,020
	491.03-982.04	125	3,7±1,3	0,023*
	982.05-2,455.10	110	3,9±1,3†	0,088†
	2,455.11-4,910.20	31	4,0±0,9*	
	≥4,910.21	19	3,6±1,5	
Escolaridade				<0,001
	Pós-graduação	79	3,8±1,1‡§	0,019‡
	Graduação	139	3,9±1,3¶&	0,012§
	Ensino médio completo/ incompleto	150	3,4±1,3‡¶#	0,001¶
	Fundamental completo/ incompleto	22	2,8±1,7§&#	<0,001&
Buscou/recebeu informação relacionada a CaP				0,041#
	Não	178	3,6±1,4	0,651
	Sim	213	3,7±1,3	
Profissional da saúde				
	Não	336	3,6±1,4	0,182
	Sim	56	3,9±1,0	
História de câncer familiar				
	Não	186	3,6±1,3	0,184
	Sim	204	3,7±1,4	

* Comparação entre as faixas de renda US$ 0 a US$ 491.02 e US$ 2,455.11 a US$ 4,910.20

† comparação entre as faixas de renda US$ 0 a US$ 491.02 e US$ 982.05 a US$ 2,455.10

‡ comparação entre escolaridade pós-graduação e ensino médio completo/incompleto

§ comparação entre escolaridade pós-graduação e fundamental completo/incompleto

¶ comparação entre escolaridade graduação e ensino médio completo/incompleto

& comparação entre escolaridade graduação e fundamental completo/incompleto

# comparação entre escolaridade ensino médio completo/incompleto e fundamental completo/incompleto.

DP: desvio-padrão; CaP: câncer de próstata.

### Conhecimento específico

Mais de 50% responderam que os tipos mais comuns de câncer eram próstata, em homens, e mama, em mulheres. Além disso, os fatores de risco mais frequentes relatados para CaP foram história familiar positiva (28,7%), idade (16,9%), tabagismo (13,3%), não sei (9,9%) e consumo de álcool (8,1%). As respostas menos frequentes incluíram obesidade (4,3%) e etnia (2,8%).

Os fatores mais relatados relacionados a melhor desfecho para CaP foram fazer exames de sangue de rotina (29,8%), exercícios físicos (24,5%), comer frutas e legumes (17,4%), não sei (12,1%) e controle do peso (9,5%).

Cerca de 82,9% responderam que deveriam ser submetidos a rastreamento para CaP, mesmo quando assintomáticos. Algumas diferenças foram observadas entre as faixas etárias. Mais especificamente, supondo um cenário assintomático, 70% dos sujeitos com ≤50 anos acreditavam que deveriam procurar assistência médica antes dos 50 anos de idade, enquanto 7,2% da mesma faixa etária acreditavam que deveriam procurar entre 30 e 50 anos. Em contraste, a proporção de respostas para as mesmas perguntas foi de 57,8% e 24,4% entre aqueles com >50 anos (p=0,008).

A maioria dos indivíduos (66,6%) respondeu que deveria realizar o rastreamento anualmente, enquanto 2% acreditavam que não precisavam de acompanhamento regular, pois a avaliação inicial seria suficiente. Em relação às possíveis ferramentas de diagnóstico para CaP, as respostas mais frequentes foram toque retal (44,5%), exame de sangue/PSA (29,7%) e ultrassom de próstata (16,4%).

### Comportamento masculino de saúde em relação a exames de rastreamento para câncer de próstata

A maioria dos participantes relatou nunca ter buscado rastreamento para CaP (68,3%), seguida por 16,3% que relataram ter consultado recentemente um médico para exames relacionados à próstata. A maioria dos homens relatou nunca ter feito dosagem de PSA (72%) ou toque retal (85%). Em uma subanálise, os participantes acima de 50 anos de idade relataram com maior frequência nunca terem buscado rastreamento (78,2% *versus* 3,4%, para >50 anos e ≤50 anos; p<0,001) (Tabela 4).

**Tabela 4:** Análise das respostas de acordo com as características demográficas, em relação à última vez em que os participantes homens buscaram rastreamento para câncer de próstata (pergunta 19 do apêndice 1)

Variável		Nunca (155/227)	>5 anos (5/227)	3-5 anos (6/227)	1-2 anos (24/227)	Este ano (37/227)	Valor de p
Idade, anos							<0,001
	≤50	3,4	3,4	6,9	44,8	41,4	
	>50	78,2	2,0	2,0	5,6	12,2	
Renda, US$							0,145
	0-491.02	76,9	1,5	3,1	4,6	13,8	
	491.03-982.04	75,4	1,4	2,9	13,0	7,2	
	982.05-2,455.10	61,7	3,3	3,3	10,0	21,7	
	2,455.11-4,910.20	40,0	5,0	0,0	25,0	30,0	
	≥4,910.21	63,6	0,0	0,0	9,1	27,3	
Escolaridade							0,077
	Pós-graduação	65,8	2,6	0,0	7,9	23,7	
	Graduação	63,0	2,5	2,5	8,6	23,5	
	Ensino médio completo	78,9	1,4	4,2	9,9	5,6	
	Ensino médio incompleto	60,0	0,0	5,0	15,0	20,0	
	Fundamental completo	100,0	0,0	0,0	0,0	0,0	
	Fundamental incompleto	58,3	8,3	0,0	33,3	0,0	

Resultados expressos como %.

Entre as razões para nunca ter realizado toque retal, as mais frequentes foram não tenho idade suficiente (63,5%), faria, sem nenhum problema (38,6%) e não é necessário (20,1%). Entre homens mais velhos, 75% responderam que o próprio médico decidiu não fazer.

## DISCUSSÃO

O escore médio do estudo foi 3,4, refletindo provavelmente um conhecimento insuficiente da população considerada. Além disso, pode sugerir problema na efetividade da campanha educacional, especialmente entre aqueles com a idade apropriada para rastreamento para CaP.

Quase metade dos participantes relatou nunca ter tido contato prévio com informações sobre CaP, especialmente os mais velhos. O fato de que mais participantes mais jovens usam a internet e as mídias sociais como ferramenta de informação, enquanto os participantes mais velhos dependem mais de uma abordagem por profissional da saúde (menos presente na rotina diária), poderia explicar parcialmente isso. Aumentar o contato entre o profissional da saúde e os pacientes mais velhos por meio de diferentes plataformas, como aplicativos ou telemedicina, pode ser uma opção.

Um achado diferente do estudo, no entanto, é que, entre os 178 participantes que responderam que nunca tiveram contato com informações sobre rastreamento para CaP, 162 responderam toque retal ou PSA como as principais ferramentas diagnósticas para CaP. Além disso, o escore médio entre os participantes com ou sem contato prévio com informações sobre CaP foi semelhante. Portanto, as respostas desafiaram as suspeitas anteriores, citadas no início da discussão, de inefetividade na divulgação de informações ao público brasileiro. Alguns participantes talvez não tenham informações suficientes para se sentirem confortáveis quanto ao conhecimento sobre a doença, o que é essencial em uma decisão compartilhada, considerando os benefícios e os riscos do rastreamento para CaP. Uma população com consciência mais consistente teria melhor adesão às recomendações.

No cenário brasileiro, poucos estudos semelhantes avaliaram esse assunto. Em estudo realizado por Ribeiro et al., incluindo 30 homens, a proporção de entrevistados que realizaram PSA foi maior que a presente.^(17)^ No entanto, 80% dos pacientes do estudo de Ribeiro et al., tinham história pessoal de câncer, impondo uma diferença consistente nas características amostrais. Por outro lado, 40% da amostra não tinha conhecimento do rastreamento de CaP, enquanto nesta amostra foram apenas 17%.

Em outro estudo envolvendo 160 indivíduos,^(18)^ 63,8% relataram PSA e toque retal como ferramentas de diagnóstico para CaP (semelhante a 74,2% deste estudo); 40,6% consideraram necessário o rastreamento anual para CaP (inferior a 66,6% deste estudo).

Estudos anteriores incluíram apenas participantes masculinos e apresentaram diferentes composições de questionários, dificultando as comparações com o presente estudo. A população da cidade de São Paulo tem os maiores níveis de escolaridade e renda entre os estados brasileiros, o que se correlaciona com maior conscientização sobre CaP.^(19)^ Ainda, a população deste estudo apresenta algumas lacunas de conhecimento, como poucos participantes identificando a origem étnica como fator de risco para CaP. Essas lacunas identificadas poderiam ser abordadas em futuras campanhas educativas.^(2,20)^

Segundo Allen et al.,^(21)^ em uma relação estável, as mulheres têm papel essencial na busca e disseminação de informações relacionadas ao CaP para seus parceiros. Por isso, é fundamental avaliar a conscientização das mulheres sobre CaP para entender melhor a adesão às recomendações de rastreamento. Entre os participantes do estudo, uma maior proporção de mulheres não tinha buscado informações sobre CaP, o que é uma questão potencial para melhorar os resultados das campanhas da SBU.^(13)^

No cenário internacional, há vários estudos sobre este assunto. O estudo de Burkina Faso demonstrou conhecimento insuficiente sobre CaP, pois 62% dos participantes nunca tinham ouvido os termos “próstata” ou “câncer de próstata”.^(2)^ Esses achados foram semelhantes em outros estudos da Nigéria.^(22,23)^

Em uma pesquisa internacional com desenho semelhante, incluindo países europeus e os Estados Unidos, demonstrou maiores níveis de conhecimento em comparação com os estudos dos países africanos. Especialmente nos Estados Unidos, 97% dos participantes tinham conhecimento do CaP.^(24)^ No entanto, 50% dos participantes desconheciam as ferramentas diagnósticas para CaP, em comparação com 2,4% desta amostra estudada. Além disso, 1% dos participantes da pesquisa internacional não sabiam que a doença poderia ser assintomática, ao passo que 82,9% dos participantes desta pesquisa sabiam que deveriam procurar esclarecer o diagnóstico de CaP, independente dos sintomas. Da mesma forma, este estudo e a pesquisa internacional relataram maior conscientização sobre o câncer de mama do que de próstata, sugerindo a influência de campanhas educativas para conscientização do público, já que os programas educacionais sobre câncer de mama estão mais estabelecidos. Além disso, ambos os estudos demonstraram percentuais semelhantes em fatores de risco correlacionados ao CaP – idade e história familiar positiva foram mencionados em 73% e 44% das respostas, contra 28,7% e 16,9% das respostas deste estudo, respectivamente. Etnia não foi respondida com frequência em nenhum dos estudos.

O estudo atual avaliou indiretamente a conscientização sobre CaP, perguntando sobre o tipo mais incidente de câncer entre homens e mulheres, tendo os participantes mais frequentemente respondido ser o de mama o de maior incidência comparado ao CaP.

Os diferentes níveis de conhecimento entre este estudo e a pesquisa internacional levantam questionamentos sobre recomendações e adesão ao rastreamento. A população paulistana sabe o suficiente para entender os riscos e os benefícios do rastreamento para CaP para uma decisão individual, como recomenda a USPSTF? Nos Estados Unidos, os achados demonstram menor busca por PSA, seguindo as recomendações nacionais, o que pode ser benéfico na redução de biópsias desnecessárias e tratamentos invasivos.^(25)^ No entanto, evidências de estudos atuais sugerem que alguns indivíduos com maior risco de desenvolver CaP já estão sendo menos avaliados.^(26,27)^ A possibilidade é que essa população desconheça alguns componentes importantes da doença para decidir se teria mais benefícios ou não, passando por um programa de rastreamento.

Pazeto et al., demonstraram que a maioria dos participantes com menos de 40 anos, submetidos à dosagem de PSA, só o fez como parte de *check-up* de saúde, já que não tinham indicação clara para isso.^(28)^ Portanto, muitos participantes não buscam especificamente rastreamento para CaP, mas provavelmente o fazem por recomendação de seu profissional de saúde ou como *check-up* geral de rotina de saúde.^(28)^ Assim, pode ser desafiador estabelecer uma decisão individual como recomendação para rastreamento para CaP, se os indivíduos nem sequer sabem o que estão fazendo.

Uma das limitações do estudo é que a população recrutada pode não ser totalmente representativa da população-alvo. Por exemplo, apenas 3,3% da população estudada não tinha ensino fundamental completo, contra 35,03% da população dentro dessa classificação na cidade de São Paulo, conforme o censo de 2010.^(29)^ No entanto, neste estudo, as etnias populacionais foram semelhantes ao percentual real da população da cidade (por exemplo, grupo branco do estudo 60,9% *versus* 60,65% grupo branco da população de São Paulo, SP, Brasil).^(30)^ Além disso, o estudo abordou uma população mais jovem em comparação com a normalmente acometida pelo CaP; porém, é importante entender o conhecimento desse grupo específico para preparar estratégias para aumentar sua conscientização no futuro.

Outra limitação foi o uso de um questionário não validado, o que dificultou a comparação com estudos semelhantes. Ele não aborda diretamente o conhecimento da população relacionada às diretrizes de rastreamento da SBU, apesar de poder demonstrá-lo indiretamente, ao perguntar quando devem visitar o médico para rastrear CaP. Embora não haja um fator protetor comprovado, como está escrito no questionário (Apêndice 1), a questão avaliou o comportamento de saúde em relação ao CaP, perguntando, por exemplo, se os participantes visitavam o médico rotineiramente para exames de rastreamento para CaP, conforme recomendado pela SBU. O desenho do escore também pode causar algumas imprecisões ao interpretar os resultados, uma vez que tenta avaliar o conhecimento dos participantes de forma arbitrária.

## CONCLUSÃO

Embora a maioria dos participantes não tenha obtido um escore satisfatório, o nível de conhecimento mostrado neste estudo parece ser superior ao de outras séries populacionais. Dessa forma, sugere que a população avaliada entendia alguns conceitos essenciais ao câncer de próstata, como a importância do rastreamento e acompanhamento. Os esforços da Sociedade Brasileira de Urologia nas campanhas educativas explicam parcialmente isso. No entanto, trabalhar em alguns conceitos, como identificar fatores de risco para câncer de próstata, pode otimizar os resultados de rastreamento.

## Apêndice 1 Questionário de avaliação de conhecimento

**Table:**

1. Qual é seu sexo?	
	a. Feminino
	b. Masculino
2. Qual é sua idade? ____________________	
3. Qual é sua renda mensal?	
	a. ≥ $ 4,910.21
	b. $ 2,455.11 a $ 4,910.20
	c. $ 982.05 a $ 2,455.10
	d. $ 491.03 a $ 982.04
	e. $ 0 a $ 491.02
4. Qual é sua escolaridade?	
	a. Fundamental incompleto
	b. Fundamental completo
	c. Ensino Médio incompleto
	d. Ensino Médio completo
	e. Graduação
	f. Pós-graduação
5. Qual é seu estado civil?	
	a. Solteiro/a
	b. Casado/a
	c. Divorciado/a
	d. União estável
	e. Viúvo/a
6. Qual é sua raça?	
	a. Branca
	b. Parda
	c. Mulata
	d. Asiática
	e. Negra
	f. Outra: __________________
7. Você é profissional da saúde?	
	a. Sim
	b. Não
7A. Se sim, em que área da saúde?	
	a. Medicina
	b. Fisioterapia
	c. Terapia Ocupacional
	d. Educação Física
	e. Farmácia
	f. Fonoaudiologia
	g. Nutrição
	h. Odontologia
	i. Psicologia
	j. Quiropraxia
	k. Outra: __________________
8. Já recebeu diagnóstico de qualquer tipo de câncer?	
	a. Sim
	b. Não
8A. Se sim, que tipo? ____________________	
9. Algum parente próximo (considere apenas avôs/avós, pai/mãe filhos/filhas, irmãos/irmãs) teve diagnóstico de qualquer tipo de câncer?	
	a. Sim
	b. Não
9A. Se sim, que tipo de câncer? ____________________	
10. Qual é o tipo de câncer mais comum em homens?	
	a. Câncer de boca
	b. Câncer de esôfago
	c. Câncer gástrico
	d. Câncer de fígado
	e. Câncer de intestino
	f. Câncer de mama
	g. Câncer de pâncreas
	h. Câncer de pele*
	i. Câncer de pênis
	j. Câncer de próstata
	k. Câncer de pulmão
	l. Câncer de rim
	m. Não sei a resposta
	n. Outro: __________________
	*Resposta correta: câncer de pele (h)
11. Qual é o tipo de câncer mais comum em mulheres?	
	a. Câncer de boca
	b. Câncer de esôfago
	c. Câncer gástrico
	d. Câncer de fígado
	e. Câncer de intestino
	f. Câncer de mama
	g. Câncer de pâncreas
	h. Câncer de pele*
	i. Câncer de pênis
	j. Câncer de próstata
	k. Câncer de pulmão
	l. Câncer de rim
	m. Não sei a resposta
	n. Outro: __________________
	*Resposta correta: câncer de pele (h)
12. Alguma vez buscou ou recebeu informações sobre câncer de próstata?	
	a. Sim
	b. Não
12A. Se sim, onde buscou ou recebeu informações sobre câncer de próstata? (pode marcar mais de uma opção)	
	a. Escola/universidade
	b. Local de trabalho
	c. Igreja/culto religioso
	d. Jornal/revista
	e. Internet/mídia social
	f. Médico/outro profissional da saúde
	g. Amigos
	h. Família
	i. Outro: __________________
13. O câncer de próstata afeta quais gêneros?	
	a. Ambos os gêneros
	b. Masculino*
	c. Feminino
	d. Não sei a resposta
	*Resposta correta: masculino (b)
14. Quais dos fatores abaixo são fatores de risco para câncer de próstata? (pode marcar mais de uma opção)	
	a. História familiar de câncer*
	b. Estresse
	c. Consumo de bebidas álcoolicas
	d. Falta de higiene
	e. Idade*
	f. Masturbação
	g. Faixa socioeconômica
	h. Obesidade*
	i. Poluição
	j. Raça/etnia*
	k. Relações sexuais frequentes
	l. Tabagismo (fumar)
	m. Não sei a resposta
	n. Outro: __________________
	*Respostas corretas: história familiar de câncer (a); idade (e); obesidade (h); raça/etnia (j)
15. Quais dos fatores abaixo podem prevenir ou diminuir o risco de câncer de próstata? (pode marcar mais de uma opção)	
	a. Comer carne vermelha toda semana
	b. Comer frutas e legumes*
	c. Exercícios físicos*
	d. Exames de sangue de físicos*
	e. Controle de peso*
	f. Não guardar o telefone celular no bolso
	g. Evitar muitas relações sexuais
	h. Não se masturbar
	i. Usar protetor solar
	j. Não sei a resposta
	k. Outro: __________________
	*Respostas corretas: comer frutas e legumes (b); exercícios físicos (c); fazer exames de sangue de rotina (d); controle de peso (e)
16. Quando deve procurar assistência médica relacionada ao câncer de próstata?	
	a. Somente quando apresentar sintomas relacionados (exemplo: sangue na urina)
	b. Assintomático, entre 30 e 40 anos
	c. Assintomático, entre 41 e 50 anos*
	d. Assintomático, entre 51 e 60 anos
	f. Assintomático, entre 61 e 70 anos
	g. Não sei a resposta
	h. Outro: __________________
	*Resposta correta: assintomático, entre 41 e 50 anos (c)
17. Com que frequência você deve retornar ao médico para monitorar câncer de próstata?	
	a. A cada 2 anos
	b. A cada 3 anos
	c. A cada 4 anos
	d. A cada 5 anos
	e. Anualmente (todos os anos)*
	f. Não há necessidade de retornar se os exames forem normais
	g. Não sei a resposta
	h. Outro: __________________
	*Resposta correta: anualmente (e)
18. Quais exames são necessários na investigação inicial para câncer de próstata? (pode marcar mais de uma opção)	
	a. Toque retal*
	b. Ressonância magnética
	c. Exame de sangue/PSA*
	d. Exame de urina
	e. Exame ósseo
	f. Ultrassom de próstata
	g. Não sei a resposta
	*Respostas corretas: toque retal (a); exame de sangue/PSA (c)
19. PERGUNTA APENAS PARA OS HOMENS – Quando foi a última vez que foi a um médico para exames relacionados à próstata?	
	a. Este ano
	b. Há 1 ano
	c. Há 2 anos
	d. Há 3 anos
	e. Há 4 anos
	f. Há 5 anos
	g. Há mais de 5 anos
	h. Nunca
20. PERGUNTA APENAS PARA HOMENS – Já fez exame de sangue para câncer de próstata? (dosagem de PSA – antígeno prostático específico)?	
	a. Sim
	b. Não
21. PERGUNTA APENAS PARA HOMENS – Você já fez exame de toque retal?	
	a. Sim
	b. Não
21A. PERGUNTA APENAS PARA HOMENS – Caso não tenha feito o exame de toque retal, por que não o fez? (pode marcar mais de uma opção)	
	a. Interfere na anatomia/fisiologia anal ou retal
	b. Interfere na potência sexual
	c. Interfere na sexualidade (interesse sexual)
	d. É doloroso
	e. Médico não solicitou
	f. Medo
	g. Não tenho idade suficiente
	h. Não é necessário
	i. Não tenho preocupação. Faria sem nenhum problema
